# Long-term agricultural reuse of treated wastewater and sewage sludge: developing a Time to Critical Content Index for metal species

**DOI:** 10.1007/s10661-024-12999-z

**Published:** 2024-08-24

**Authors:** Patricia Merdy, Rabia Cherfouh, Yves Lucas

**Affiliations:** 1 Aix Marseille Université, CNRS, IM2NP, Université de Toulon, Toulon, France; 2 Laboratoire d’Ecologie, Biotechnologie et Santé (LEBS), Université Mouloud MAMMERI, PB 17, Tizi-Ouzou, RP 15000 Algeria

**Keywords:** Potentially toxic metal speciation, Risk index, Sewage sludge, Soil quality, Sustainable agriculture, Treated wastewater

## Abstract

This study evaluates the sustainability of spreading wastewater or sewage sludge on agricultural land, balancing benefits with contamination risks. Conventional ecological risk indices often fail to address the long-term accumulation of metals in soils. We investigate the feasibility of spreading based on current knowledge of potentially contaminating metals and their behavior in soil. We analyzed the speciation of metals (Ag, Cd, Co, Cr, Cu, Ni, Pb, Ti, Zn) through sequential extraction in sludge, treated wastewater, and soils after 14 years of application of sewage sludge and treated wastewater issued from an Algerian wastewater treatment plant. We introduce a Time to Critical Content Index (TCCI) that calculates the time required to reach critical levels of potentially mobile metals, considering total metal content and speciation. The TCCI takes into account product knowledge, soil characteristics, metal behavior, ecological/toxicological thresholds, and regulations. Applied to our case study, the TCCI indicates that spreading sewage sludge can continue despite metal contents exceeding regulatory ceiling values. The index serves as a precautionary measure, adaptable to evolving knowledge, providing a comprehensive framework for sustainable agricultural practices.

**Graphical Abstract:**

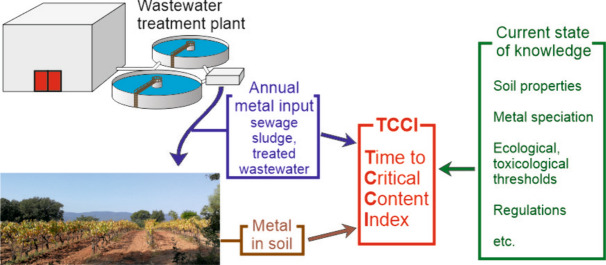

**Supplementary Information:**

The online version contains supplementary material available at 10.1007/s10661-024-12999-z.

## Introduction

The pressure on soil productivity, the search for a reduction in commercial mineral fertilizers, and the push for a circular economy are driving the development of the agricultural use of treated wastewater (TWW) and associated sewage sludge (SS). Such practices, however, must preserve soil fertility and avoid soil contamination in the long term, as TWW or SS is likely to be a source of significant quantities of contaminating substances, heavy metals, or organic compounds (Buta et al., 2001; Hechmi et al., 2023). The necessity of considering these risks has led to numerous studies over the past 50 years and prompted public authorities in many countries to establish guidelines for the use of TWW and SS based on their metal content and the characteristics of the soils on which they are spread (Chen et al., 2018; Latosińska et al., 2021).

Until the 2010s, research efforts primarily focused on the agronomic consequences of using TWW or SS and on contamination by heavy metals (Hechmi et al., 2020; Singh & Agrawal, 2008). Since then, most studies shifted their focus to organic compounds, such as persistent organic pollutants (POPs) or microplastics (Buta et al., 2001).

Regarding heavy metals, there are still many questions that need to be answered. Public regulations currently consider the total metal content of TWW and dehydrated SS (DSS) and, in certain cases, the pH of soils. If the metal content exceeds the regulatory value, agricultural spreading of TWW or DSS may be restricted, even if the metal is not bioavailable. The concept of metal bioavailability has long been considered in the scientific literature (Kim et al., 2007; Yu et al., 2024), but it has not yet been translated into public regulations due to the complexity of its evaluation. There are many techniques to assess the bioavailability of a metal in soil, such as plant uptake, chemical-based extractions on bulk or rhizosphere soil, isotopic dilution techniques, or diffusive gradients in thin films. However, these methods vary in the time they consume. Among them, the five-step chemical sequential extraction procedure by Tessier et al. (1979) remains the most widely used. This extraction divides metals into five fractions according to their lability conditions: (1) an easily exchangeable fraction (*F*_EX_); (2) a strongly adsorbed fraction (*F*_AC_); bound mainly to carbonates or, if present, to part of the Fe-sulfides; (3) a fraction bound mainly to Fe and Mn (hydr)oxides (*F*_RED_); (4) a fraction bound mainly to organic matter or sulfides (*F*_OX_); and (5) a residual fraction (*F*_RES_), extracted with aqua regia assisted by micro-waves. This fractionation, interpreted as chemical speciation (Vilar et al., 2005), has been the basis of several ecological risk indices that can be applied to soils or sediments. The main ones are listed in Table 1.

**Table 1:** Some ecological risk indices based on sequential extraction

Name and reference	Index	Range of variation low risk high risk
Risk Assessment Code (RAC) (Jain, 2004)	$\mathrm{RAC}=\frac{{F}_{\mathrm{EX}}+{F}_{\mathrm{AC}}}{{F}_{\mathrm{TOT}}}$	0 ➜ 1
Individual Contamination Factor (ICF) (Ikem et al., 2003)	$\mathrm{ICF}=\frac{{F}_{\mathrm{EX}}+{F}_{\mathrm{AC}}+{F}_{\mathrm{RED}}+{F}_{\mathrm{OX}}}{{F}_{\mathrm{RES}}}$	0 ➜∞
Reduced Partition Index (*I*_R_) (Han et al., 2003)	${I}_{\mathrm{R}}=\frac{{F}_{\mathrm{EX}}+{{2}^{n}F}_{\mathrm{AC}}+{{3}^{n}F}_{\mathrm{RED}}{+{4}^{n}F}_{\mathrm{OX}}{+{5}^{n}F}_{\mathrm{RES}}}{{5}^{n}{F}_{\mathrm{TOT}}}$ with *n* chosen from 1 to 2	1 ➜ 0.04 for *n* = 2
Individual Ecological Risk (IER) index (Tytla, 2020)	$\mathrm{IER}=\frac{{F}_{\mathrm{EX}}+{F}_{\mathrm{AC}}+{F}_{\mathrm{RED}}}{{F}_{\mathrm{OX}}{+F}_{\mathrm{RES}}}$	0 ➜ ∞

In all these indices, the terms in the numerator represent fractions that are easily soluble in the rhizosphere and can enter living organisms. The *I*_R_ index takes into account all the fractions by assigning each a weight according to its potential liability. All these indices, however, remain weighted by the total metal content or by the less mobile fractions, thus representing a relative binding intensity of the metal. Therefore, two materials can have the same index value for high or negligible values of the total metal, while the associated risk is not the same. For example, for a given metal, a sludge having 5 mg kg^−1^ of total metal with 1 mg kg^−1^ in each fraction has the same indices as a sludge having 1000 mg kg^−1^ of total metal with 200 mg kg^−1^ in each fraction, while the risk associated with their use is not the same: they will have, respectively, 2 and 400 mg kg^−1^ in the two most labile fractions (*F*_EX_ + *F*_AC_). Integrated indices have also been developed, which consider all the metals present to assess the overall risk at a given location (Weissmannová & Pavlovský, 2017). However, these indices are not suitable in cases where the risk associated with each individual metal must be assessed.

To predict the risk associated with a specific metal speciation, it must also be considered that its mobility and bioavailability depend on the characteristics of the soil and the rhizosphere (Feng et al., 2005), as well as their potential evolution over time. According to Römkens et al. (2004), the dissolved concentration and the free metal ion activity in the soil, with regard to a reactive metal content, can be predicted based on factors such as soil pH, the content of soil organic matter, clay, amorphous Fe and Al oxides, the concentration of Ca and dissolved organic carbon in the soil solution, and CEC. However, obtaining these data for all plots where TWW or SS is to be applied can be challenging, costly, and time-consuming. Therefore, it is necessary to use the best approximate model possible from the available data (Kopstsik & Koptsik, 2022).

Moreover, studies are rare that integrate the speciation of all the potentially problematic metals both in the spread products (TWW or DSS) and in soil with regard to critical loads (Khadhar et al., 2020).

In this context, the present study aims to address the lack of an index that allows for the assessment of the long-term risk associated with the application of agronomic products. This study focused on agricultural soils developed under a semi-arid Mediterranean climate (Algeria). After long-term application (14 years) of municipal TWW or DSS under three different types of application (TWW only, DSS only, both TWW and DSS), these soils were analyzed to evaluate the impact of these applications. The initial part of the research, which focused on agronomical properties, was published previously by Cherfouh et al. (2018). In the present study, we quantified the input of Ag, Cd, Co, Cr, Cu, Ni, Pb, Ti, and Zn and investigated their behavior in soils using sequential chemical extraction. Our data serve as a basis for establishing a risk index for each metal input, based on a precautionary principle and adaptable to the data available for the soils in question.

## Materials and methods

### *Study area*

**Fig. 1:**
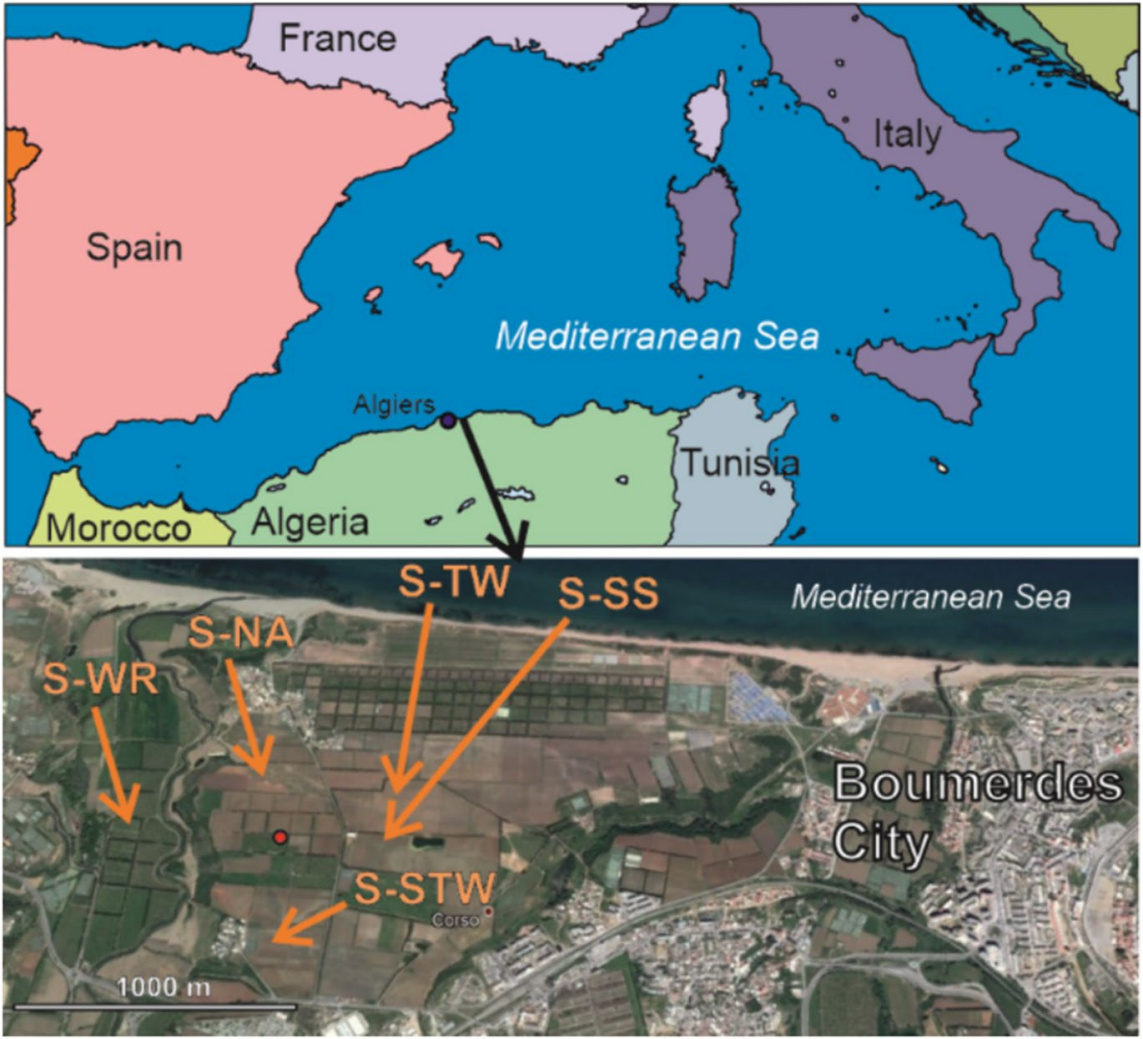
Location of the sampling sites in the Boumerdes region, Northern Algeria. The red point indicates N 36° 45′ 34.56″, E 3° 25′ 24.96″. View from Google Earth, 2017

The study area is located in northern Algeria, within the territory of Corso city, in the district of Boumerdes (Fig. 1). The geological substrate consists of compact red sand overlaying older marl. The region’s climate is Mediterranean, with an annual average rainfall of 650 mm. The use of DSS and TWW from the Boumerdes city wastewater treatment plant (WWTP) began in 2002 on agricultural land cultivated for table grapes. The sludge is applied once a year at an estimated rate of 15 to 20 tons per hectare, while drip irrigation with TWW is practiced from May to October to compensate for water shortages. NPK fertilizers are commonly used, but applied quantities applied are unknown to the farmers; more details can be found in Cherfouh et al., (2018, 2022). The map (Fig. 1) shows the sampling sites, located about 1 km from the sea coast.

Soils are classified as calcisols (IUSS, 2015) developed on Miocene molasses (Magne & Raymond, 1974). Detailed characteristics of the sampled soils are given in a previous publication (Cherfouh et al., 2018). Soils are loam (S-SS), silt loam (S-TW, S-NA), or clay loam (S-STW, S-WR), with a pH slightly alkaline to neutral (6.9 to 7.8). Their main characteristics are summarized in Table 2.

**Table 2:** Main soil characteristics. *ρ*_*d*_, apparent density; *SOC*, soil organic carbon. Soil texture classes from FAO, 2008

Depth (cm)	SOC (% w/w)	pH	CEC (cmol_c_ kg^−1^)	Clay	Silt	Sand	*ρ*_*d*_ (g cm^−3^)	
				(%)				
S-SS (soil receiving DSS)								
0–10	2.3	7.7	16.7	15.6	52.6	31.9	1.1	Silt loam
10–25	2.2	7.5	16.4	13.5	48.6	37.7	1.3	loam
25–40	1.5	7.5	14.5	22.5	36.0	41.5	1.3	loam
S-TW (soil receiving TWW)								
0–10	1.3	7.3	11.8	18.6	52.8	28.5	1.3	Silt loam
10–25	1.3	7.3	12.3	19.3	53.3	26.9	1.4	Silt loam
25–40	0.9	7.0	10.4	25.6	47.8	26.4	1.5	Silt loam
S-STW (soil receiving both DSS and TWW)								
0–10	1.6	7. 6	19.9	40.0	25.7	34.3	1.3	Clay
10–25	1.3	7.5	19.7	36.7	28.3	35.1	1.6	Clay loam
25–40	0.8	7.6	18.6	34.7	35.0	30.3	1.5	Clay loam
S-WR (soil receiving river water)								
0–10	4.7	7.8	19.4	31.0	46.0	23.0	1.3	Clay loam
10–25	4.5	7.8	19.7	30.0	36.0	34.0	1.3	Clay loam
25–40	4.2	7.5	19.5	31.0	46.0	20.0	1.4	Silt loam
S-NA (reference soil)								
0–10	0.9	7.1	9.6	7.3	55.5	37.2	1.3	Silt loam
10–25	1.0	7.0	10.4	7.7	60.7	34.4	1.4	Silt loam
25–40	0.6	6.9	9.3	21.6	52.8	25.6	1.5	Silt loam

### Sampling

We sampled soils from five representative vineyards located in Fig. 1, which were similar in terms of parent rock, soil type, topographical situation, and farming techniques. They differed only in the use of SS amendment, TWW irrigation, and river water applications. We collected soil receiving dehydrated sewage sludge (S-SS), soil irrigated with TWW (S-TW), soil receiving both sludge and TWW (S-STW), and reference soil that received neither sludge nor TWW (S-NA). We also collected soil irrigated with river water (S-WR). Sampling was performed in April 2013 at three depths (P1 0–10 cm; P2 10–25 cm; P3 25–40 cm). These depths were chosen as the best compromise to appreciate variations with depth while maintaining a reasonable number of samples. All soil samples were taken within 1 month to minimize seasonal variations.

At each site and for each depth, five cores were sampled at the corners and at the center of a 1-m square plot using an auger. A composite soil sample was generated by thoroughly mixing the five subsamples from each depth. Samples were air-dried and sieved to 2 mm. Aliquots of the < 2 mm soil fraction were ground in an agate mortar and stored in polyethylene containers at 4 °C until analysis. Sewage sludge was sampled at the Boumerdes WWTP by taking five samples on the same day and mixing them to form an average sample, representing an integrated deposit over a period varying between 20 and 60 days, making a random result unlikely. Three TWW samples were collected at the Boumerdes WWTP outlet.

### Analysis

**Fig. 2:**
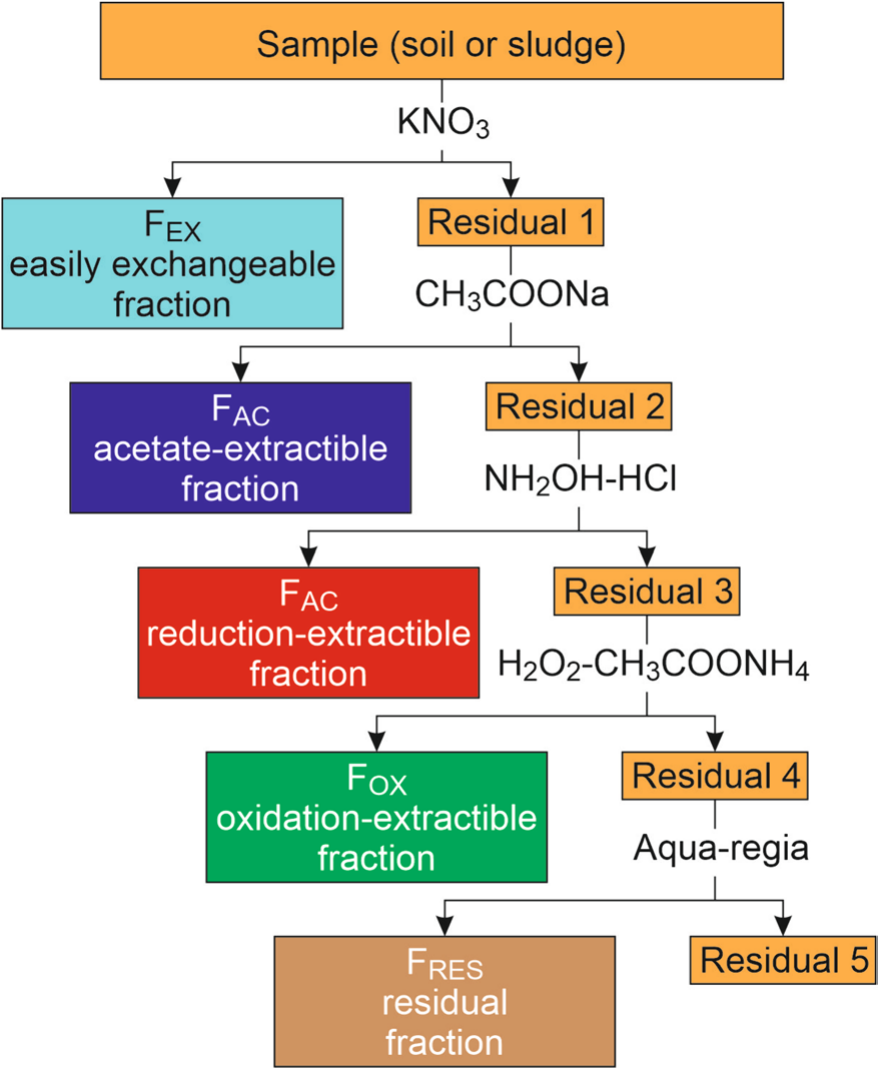
Diagram of the procedure for sequential extraction of trace elements

All solutions were prepared with high-purity water (resistivity ≈ 18 µs cm^−1^, Milli-Q Plus system, Millipore Corporation, USA) and analytical grade reagents (Merck). Sequential extraction was performed in triplicate using a five-step procedure modified from Tessier et al. (1979), according to a protocol described in Cherfouh et al. (2022), to obtain five fractions (Fig. 2). For each sequential extraction, three replicates were performed in order to meet statistical acceptance.

Requirements:

- Fraction *F*_EX_: easily exchangeable fraction; extracted using KNO_3_ 1 M
- Fraction *F*_AC_: strongly adsorbed fraction, bound mainly to carbonates or part of the Fe-sulfides, extracted using sodium acetate 1 M at pH = 5
- Fraction *F*_RED_: fraction bound mainly to Fe and Mn (hydr)oxides, extracted using hydroxylammonium chloride 0.25 mol L^−1^ at pH 1.5
- Fraction *F*_OXI_: fraction bound mainly to organic matter or sulfides, extracted using 30% H_2_O_2_ then ammonium acetate 3.2 mol L^−^.^1^ acidified at pH 2 with HNO_3_
- Fraction *F*_RES_: residual fraction extracted with aqua regia assisted by microwave

Metal analysis was performed by atomic emission spectrometry with inductively coupled plasma ICP-AES (Jobin Yvon UltraTrace 2000 equipped with a vertical plasma torch), LCE research team at Marseille University. Validation of the ICP measurements was conducted following usual analytical laboratory practice, using commercial multi-element standard (Agilent 6,610,030,600), working standards prepared with trace metal grade stock solutions, and inter-laboratory testing. The limit of reporting (LOR) for all trace elements analyzed in the extracts is provided in the Supplementary Material. Statistical analysis was conducted using the XLSTAT software (Addinsoft).

## Results and discussion

### Total metals in treated wastewater, sewage sludge, and soils

**Fig. 3:**
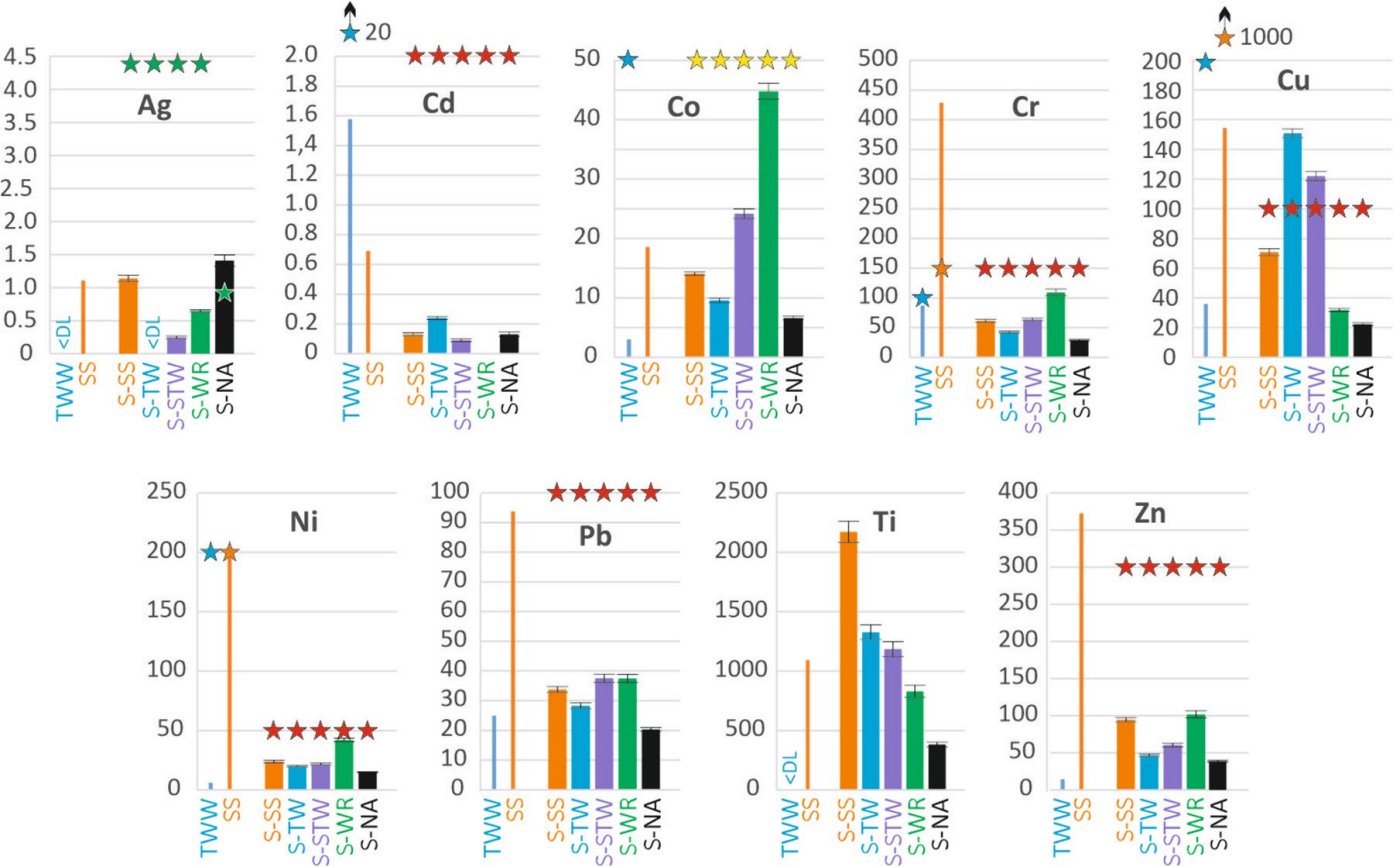
Total metal concentration in TWW (average) and total metal content in SS and in the 0–40 topsoil horizons of the studied soils. Values are in µg L^−1^ for concentration in TWW and in mg kg.^−1^ for content in SS and soils. Error bars give ± standard deviation. When existing and within the scale range, threshold values are indicated by stars: for TWW, ceiling value for long-term application (FAO, 2003); for SS, ceiling value for application (France) (JO, 1998); for soils, red stars, ceiling value to allow TWW or SS application (France) (JO, 1998); yellow stars, ecological investigation assessment level (Australia) (WA DOE, 2003); green stars, ecotoxicity level (Kolesnikov et al., 2020)

Total metal concentration in TWW and content in SS and soils are given in Fig. 3; average numerical values and standard deviations are provided in the Supplementary Material. The average coefficient of variation between the three replicates was 9.6%, and the maximum coefficient of variation was 18.0%. The ceiling values correspond to French regulations (JO, 1998) or, when not specified there, have been found in the literature. The French regulation values are close to those used in other countries, both for suspended solids (Chen et al., 2018) and for soils (Latosińska et al., 2021).

In TWW, Ag and Ti concentrations were below the limit of reporting. Cd, Co, Cu, Ni, Pb, and Zn concentrations were far below the TWW long-term application ceiling values. The Cr concentration was on average slightly lower than the ceiling value, although one of the three measured values was higher (226 µg L^−1^). Regarding SS, only Cd, Cr, Cu, Ni, Pb, and Zn have regulatory ceiling values. For all these metals, the SS content was below the ceiling value for all metals except Cr, whose content was almost three times higher than the ceiling value. A discussion on the possible origin of the studied metals (industries or cottage industries related to painting, metal working or plating, tannery, textile dyeing) can be found in Cherfouh et al. (2022).

In soils, the Cd, Pb, and Zn contents were consistently well below the permitted ceiling values for TWW or SS application (JO, 1998). The Cr and Ni contents were closer to the ceiling values. However, no observable accumulation effect was found in soils subjected to SS or TWW inputs. The Cu content was excessive, i.e., above the ceiling value, for the S-TW and S-STW soils, and slightly lower than the ceiling value for the S-SS soil, indicating that SS or TWW inputs are non-regulatory or not recommended in these soils. The high Cu content in these soils is most likely due to the common use of phytosanitary Cu compounds on the vines. There is no regulatory ceiling value for Ag, Co, and Ti. For Ag, the ecotoxicity threshold was measured at 4.4, 0.9, and 0.8 mg kg^−1^ for a pH 7.8 chernozem, a pH 6.8 arenosol, and a pH 5.8 cambisol, respectively (Kolesnikov et al., 2020). Because pH primarily influences metal mobility (VandeVoort & Arai, 2012), the value applicable to chernozem soils can be used for S-SS, S-TW, S-STW, and S-WR, while the value for arenosol soils applies to S-NA (Fig. 3). In the S-NA soil, the Ag content appeared excessive. For Co, all soil contents remained below the level corresponding to the assessment of ecological investigations (WA DOE, 2003). We found no data on toxic Ti content level in soils other than TiO_2_ nanoparticles. However, the observed Ti contents remained far below the average Ti content of the earth’s crust (4400 mg kg^−1^), which rules out a risk of Ti toxicity.

The soil irrigated with river water (S-WR) had the highest contents for Co, Cr, Ni, and Zn, but these contents were still below critical levels. This may be due to uncontrolled and untreated releases into the river from small industries such as metal surface treatment, damascene, textiles, and tanning (Cherfouh et al., 2022).

We calculated the minimum time required to accumulate the metal to a critical content (ceiling content, ecotoxicity threshold, toxic level, or else), i.e., the value indicated by a star in Fig. 3, in the 0–40 cm topsoil. The depth of 40 cm was chosen as the maximum plowing depth with the techniques used in the study area. We considered the total metal contained in SS and TWW, assuming no metal leaching out and a constant input of 200 mm year^−1^ of TWW and 30 t ha^−1^ year^−1^ of SS, as applicable. We called this minimum time the TCCI (Time to Critical Content Index); results are given in Table 3, Statement 1. The uncertainty related to the standard deviation was less than 10% for all calculated times. As noted above, critical content was already exceeded for Ag in S-SS and Cu in S-TW and S-STW. For Cr and Ni in S-SS and S-STW and for Cu in S-SS, critical content can be reached in a short time, less than 50 years, with the origin of the metal from SS.

**Table 3:** TCCI: minimum time (years) to reach critical metal content in the 0–40 cm topsoil under different statements and assuming constant TWW or DSS input. The ± value is the uncertainty related to the standard deviation on the metal content values. *aacc*, already above critical content. *F*_EX_, *F*_AC_, *F*_OXI_, *F*_RED_ fractions: refer to Fig. 2

	Ag	Cd	Co	Cr	Cu	Ni	Pb	Zn
Statement 1. Considering the total metal in TWW and DSS and applying a critical threshold to the total metal content								
S-SS	495 ± 14	457 ± 2	325 ± 0	35 ± 2	32 ± 3	22 ± 4	119 ± 9	93 ± 3
S-TW	/	3135 ± 18	/	3474 ± 97	aacc	/	8072 ± 221	/
S-STW	771 ± 4	496 ± 3	286 ± 0	41 ± 2	aacc	22 ± 2	135 ± 6	133 ± 3
Statement 2. Considering the potentially mobile fraction ((*F*_EX_ + *F*_AC_ + *F*_OXI_) for Cd, Cr, Cu, Pb, Zn and (*F*_EX_ + *F*_AC_ + *F*_OXI_ + *F*_RED_) for Ag, Co, Ni) in place of total metal								
S-SS	495 ± 14	1251 ± 3	779 ± 12	1761 ± 4	379 ± 4	322 ± 6	10,940 ± 21	1437 ± 26
S-TW	/	3510 ± 5	/	4742 ± 11	5764 ± 182	/	11,071 ± 18	/
S-STW	/	1103 ± 2	966 ± 14	1519 ± 4	406 ± 7	326 ± 11	6391 ± 10	1950 ± 12

The information obtained from the total metal concentrations and contents leads to the following conclusion. According to the cited regulations, particular attention should be paid to the Cd and Cr concentrations in the TWW and to the Cr and Ni contents in the SS. Moreover, some soils should not, due to their high Cu content, receive TWW or SS inputs. For Ni and Cr, the critical value can be reached in less than 50 years of SS amendments. However, these considerations are a rough approach for estimating the risks involved in using SS or TWW. The risk is likely overestimated, so a finer approach should be proposed that takes into account the potential mobility of metals in the SS as well as in the soils.

### Metal speciation in sewage sludge and soils

In Mediterranean soils where sludges are spread, the fractions most likely to release bioavailable metals are the *F*_EX_ and *F*_AC_ fractions, as well as the *F*_OXI_ fraction. The latter corresponds to metals bound to sulfides and organic matter, which are rapidly oxidized in a dry environment and, in the case of organic matter, at pH levels higher than 6.5 (Cherfouh et al., 2022). The *F*_RED_ fraction is likely to be poorly mobile in soils of the area which are not prone to waterlogging. The *F*_RES_ fraction is the least mobile.

**Fig. 4:**
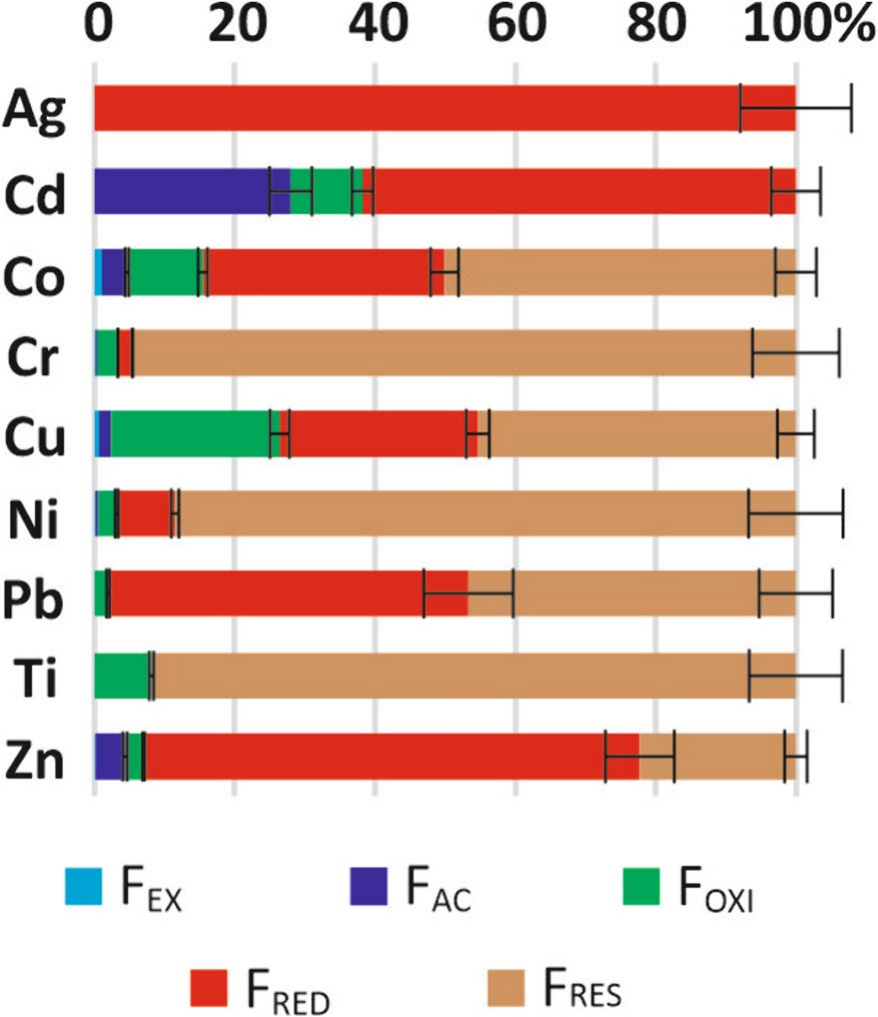
Metal speciation in sewage sludge. Error bars give ± standard deviation

The metal speciation in the sewage sludge is given in Fig. 4. For each metal except Ag, the speciation was close to that observed in four other treatment plants located in the same area (Cherfouh et al., 2018), but there were significant differences between metals. Ti, Cr, and Ni were found mostly in the residual fraction (*F*_RES_), i.e., contained in minerals, therefore poorly bioavailable (Sims & Kline, 1991). Co was also mainly found in the *F*_RES_ fraction but with a higher proportion in the *F*_OXI_ and *F*_RED_ fractions and a significant proportion (4.7%) in the (*F*_EX_ + *F*_AC_) fractions. Zn and Pb were mostly found in the *F*_RED_ fraction, as observed elsewhere (Silveira et al., 2006; Chen et al., 2008; Zufiaurre et al., 1998), with, for Zn, a non-negligible proportion (4.4%) in the (*F*_EX_ + *F*_AC_) fractions. Cu was mainly distributed between the *F*_RED_, *F*_OXI_, and *F*_RES_ fractions, with a small (*F*_EX_ + *F*_AC_) proportion (2.4%). Compared to other metals, Cu was mainly associated with the *F*_OXI_ fraction, a similar finding to that observed by other authors (Chen et al., 2018; Silveira et al., 2006; Zufiaurre et al., 1998). Cd was below the limit of reporting in the *F*_RES_ fraction. Most of it was distributed between the *F*_AC_ and *F*_RED_ fractions, with a significant part (10.3%) in the *F*_OXI_ fraction. A similar distribution has been described for sludge from the middle-south region of China (Chen et al., 2018). Ag was below the limit of reporting in all fractions except in *F*_RED_. The speciation of this metal can, however, be very different from one WWTP to another (Cherfouh et al., 2022).

**Fig. 5:**
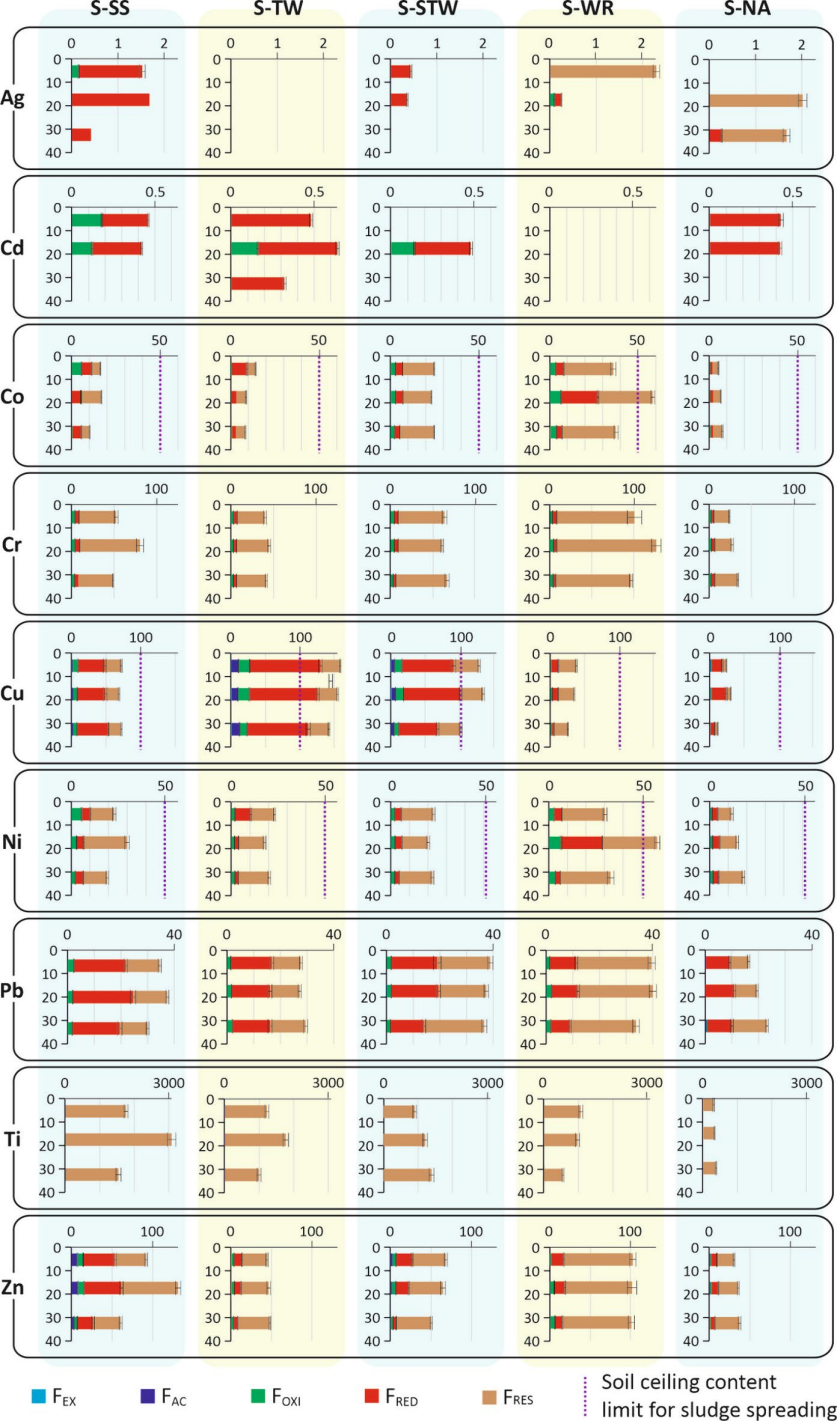
Metal speciation in the studied soils. Values in mg kg^−1^. Error bars give ± standard deviation. Ceiling content values are given when within the scale range

The metal speciation in the studied soils is given in Fig. 5. Ag speciation values were different from one soil to another, but always well below the ecotoxicology threshold (4.5 mg kg^−1^). Cd was found in the *F*_OXI_ and *F*_RED_ fractions, the latter probably poorly mobile in the studied soils, and in any case at levels well below the ceiling value (2 mg kg^−1^). Co, Cr, Ni, Pb, Ti, and Zn were found mainly in the less mobile *F*_RED_ and *F*_RES_ fractions and, excepting Co and Ni, were at levels well below the ceiling values, where they exist. Co and Ni were in the 15–20 cm horizon of the S-WR soil at levels (58.3 and 58.6 mg kg^−1^, respectively) higher than the threshold (50 mg kg^−1^) for ecological investigation. However, only 6.2 and 6.5 mg kg^−1^, respectively, were found in potentially mobile fractions (*F*_EX_ and *F*_OXI_). Total Cu was close to or higher than the ceiling content in all horizons from the S-SS, S-TW, and S-STW soils. More than 80% of Cu, however, was found in the poorly mobile *F*_RED_ and *F*_RES_ fractions, so even in soil with the highest total Cu content (S-TW), the total of the mobile fraction did not exceed 28% of the ceiling content. Since Cu-based fungicides have been widely used for a long time, especially in vineyard soils, the behavior of Cu in soils has been the subject of numerous studies. These have confirmed that the mobility and bioavailability of Cu are not related to its total content, but to the soil pH which determines the conditions of release of Cu from the *F*_RED_ fraction. At a pH higher than 6.5, this release is very low (Cornu et al., 2019).

**Fig. 6:**
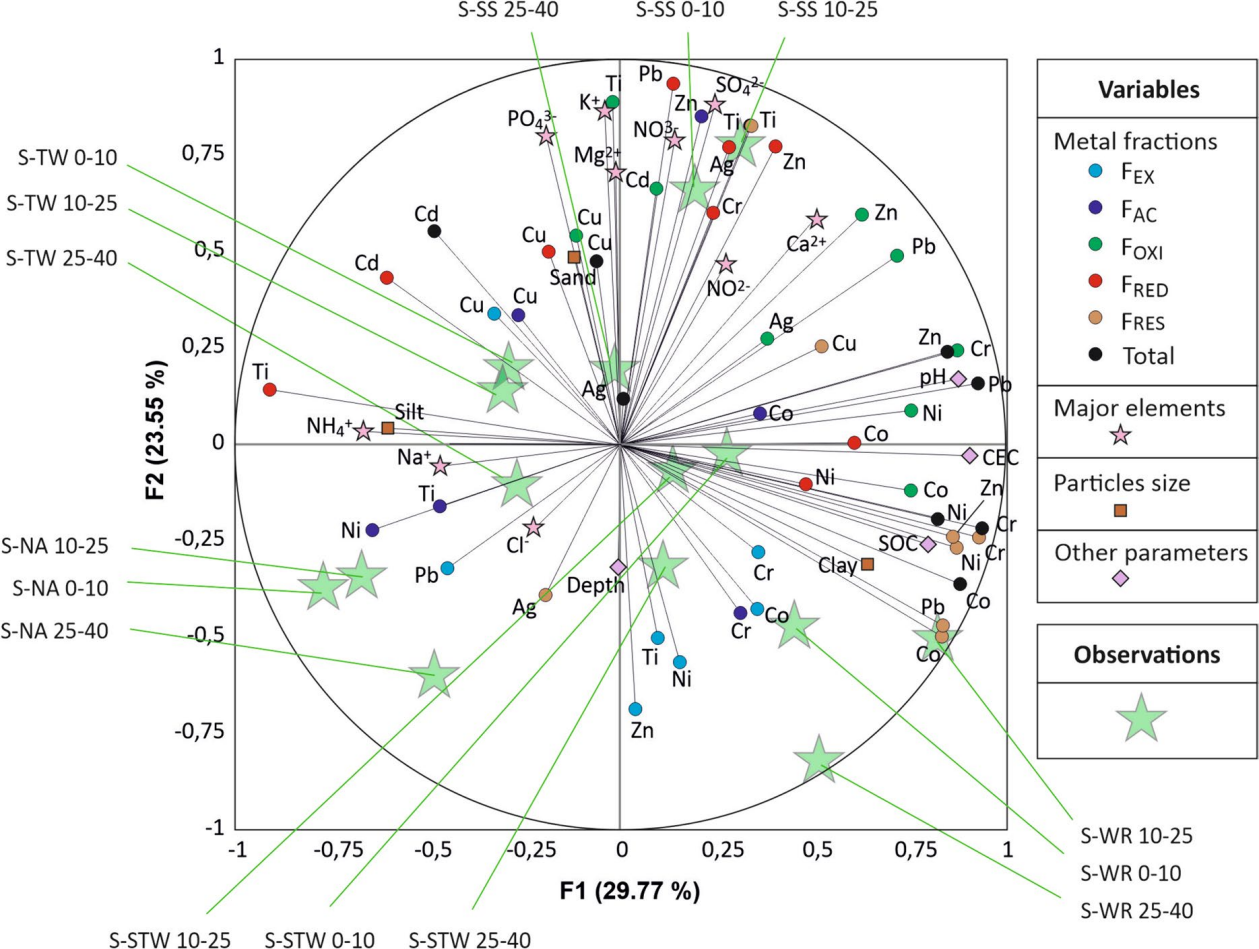
PCA biplot on the first two factorial axes for the whole set of data

Correlations between metal species are visualized through a PCA analysis (Fig. 6). The first factorial axis was primarily associated with silt content versus clay content, pH, CEC, and SOC, with these latter parameters being highly correlated with the residual fraction for all metals. The second factorial axis indicated that the easily extractable *F*_EX_ fractions for all metal except Cu were related to the S-WR soil, likely due to river water contamination by cottage industries involved in painting, metalworking and plating, tannery, and textile dyeing (Cherfouh et al., 2022).

**Fig. 7:**
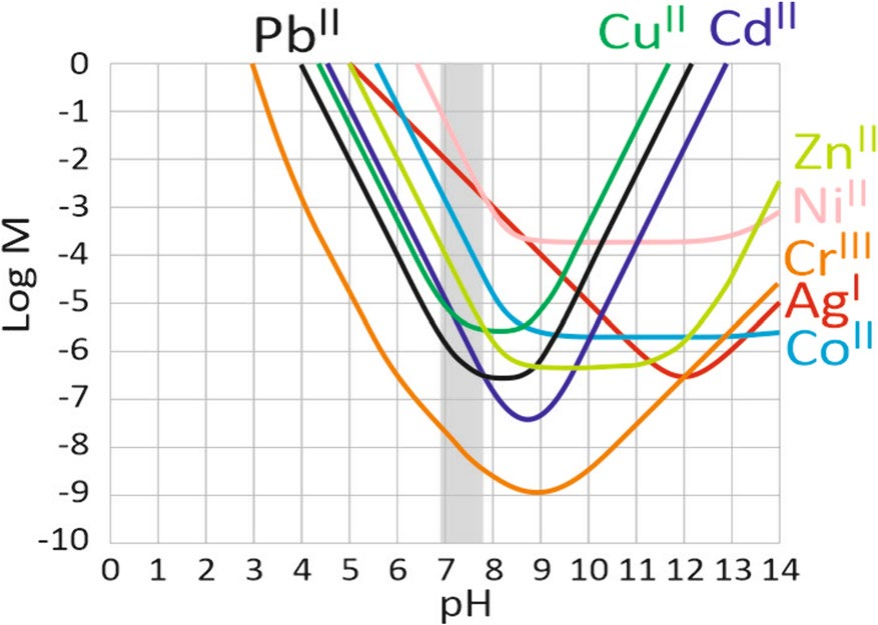
Solubility domain of the studied metals at equilibrium with their more soluble hydroxide or carbonate mineral species (Ag_2_CO_5_, CdCO_3_, CoCO_3_, Cr(OH)_3_ am, Cu(OH)_2_, Ni(OH)_2_, PbCO_3_, Zn_2_CO_3_ H_2_O), in water at equilibrium with the atmosphere. The gray area indicates the pH range of the studied soils. Calculation using the WATEQ4F database (Ball & Nordstrom, 1991)

To account for speciation data, we recalculated the TCCI for topsoil 0–40 cm, considering only the metal contained in the potentially mobile fractions. For Cd, Cr, Cu, Pb, and Zn, the *F*_RED_ and *F*_RES_ fractions can be considered immobile in non-acid soil. They are not subject to anoxic conditions, where soil organic matter is therefore not very mobile (Brun et al., 1998; Ertani et al., 2017; Koptsik & Koptsik, 2022; Kubier et al., 2019; Rutkowska et al., 2015). In the pH range of the studied soils, these metals are indeed relatively insoluble in water at equilibrium with the atmosphere (Fig. 7). For Ag, Co, and Ni, we considered that the *F*_RED_ fraction was potentially mobile, taking into account their higher solubility in the pH range of the studied soils (Fig. 6). For Co and Ni, we referred to the results of the literature (Harter, 1992; Woodward et al., 2018). For Ag, there is a lack of knowledge about its behavior. Under these considerations, there are no longer any soils with a metal content above a critical value, regardless of the metal (Table 2, Statement 2). The time required to reach a critical value by the application of TWW and/or DSS is greater than 300 years for all metals.

Therefore, taking into account the mobility conditions of metals, assessed by the study of their speciation, makes it possible to extend the possibilities of agricultural recovery of wastewater and sludge.

### The Time to Critical Content Index (TCCI)

All of these considerations lead us to propose the TCCI as a soil contamination risk index that can be adjusted according to advances in knowledge about the products being spread and the recipient soils, as well as any changes in public regulations. For the reasons explained in the introduction, this index must not be relative, weighted by the total metal content or by the less mobile fractions. We propose to evaluate the time that would lead to mobile metal species reaching a critical level determined by public regulations or scientific considerations. For a given metal, the Time to Critical Content Index (TCCI) (years) can be defined as follows:

$$\mathrm{TCCI}= \frac{\left({\mathrm{Cc}}_{\mathrm{soil}}-{\mathrm{Mc}}_{\mathrm{soil}}\right) \rho\;{S}_{D}}{\mathrm{AL}}$$

where Cc_soil_ (mg kg^−1^) is the critical content of the potentially mobile metal in the soil, Mc_soil_ (mg kg^−1^) is the average content of the potentially mobile metal over the considered soil depth, *S*_*D*_ (m) is the considered soil depth, *ρ* (kg m^−3^) is the average bulk density over the soil depth considered, and AL (mg m^−2^ year^−1^) is the annual load of potentially mobile metal. The relevant soil depth to be considered may vary site-specifically depending, for example, on the agricultural techniques or the density of deep roots. The expression of the annual load AL depends on the products that are spread on the soil. If for example both SS and TWW are spread, AL will be:

$$\mathrm{AL}={\mathrm{Mc}}_{\mathrm{SS}} {\mathrm{AL}}_{\mathrm{SS}}+{\mathrm{Mc}}_{\mathrm{TWW}} {\mathrm{AL}}_{\mathrm{TWW}}$$

where Mc_SS_ (mg kg^−1^) is the potentially mobile metal content in SS, AL_SS_ (kg m^−2^) is the annual load of SS, Mc_TWW_ (mg L^−1^) is the metal concentration in TWW, and AL_TWW_ (L m^−2^) is the annual load of TWW. We propose that the risk associated with a given input is very high for TCCI < 50, high for 50 ≤ TCCI < 150, moderate for 150 ≤ TCCI < 300, and low for 300 > TCCI (Table 4).

**Table 4:**
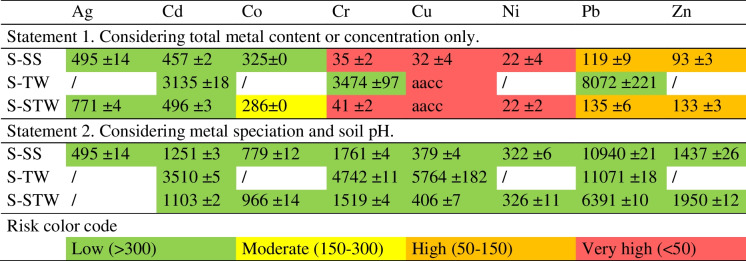
Risk assessment using the TCCI. The ± value is the uncertainty related to the standard deviation on the metal content values. aacc: already above critical content

The metal that will be considered potentially mobile, meaning likely to pass from the soil to the biota, depends on prior knowledge of the speciation of the metal in the soil and the spread products, as well as the characteristics of the soil. The minimum knowledge required is the total metal content of the soil and the input products. In our example, if only the total metal contents of soil, SS, and TWW were known, all of these total metals would be considered potentially mobile and the TCCI would correspond to the values calculated for Statement 1 in Table 2. Therefore, spreading SS and TWW would not be recommended for all three soils. Since sequential extraction and soil pH data are available, the potentially mobile metal depends on the soil characteristics. In our example, because of the soil pH, the *F*_RES_ fraction can be considered inert for all metals, and because reductive dissolution of compounds in the soil is unlikely, the *F*_RED_ fraction can be considered inert for Cd, Cr, Cu, Pb, and Zn (Fig. 6) (Yang et al., 2018). The TCCI corresponds to the values calculated for Statement 2 in Table 2, all showing a low risk: the spreading of SS and TWW on the soils can therefore be implemented. If the soil was susceptible to waterlogging, the *F*_RED_ fraction could not be considered inert for any metal. The TCCI index may be re-evaluated as new information relating to the products applied, the characteristics of the soils, or the behavior of metals becomes available.

Table 4 Risk assessment using the TCCI. The ± value is the uncertainty related to the standard deviation on the metal content values. *aacc*, already above critical content.

## Conclusions

When assessing the risk associated with the input of metals into the soil from regular additions of agricultural products, such as SS or TWW, risk indices weighted by the least mobile fractions of the metal considered are not well-suited. The cumulative input over time of the potentially mobile fractions must be related to the critical content thresholds in the soil of ecological or toxicological interest. This approach, which leads to the definition of the TCCI, has the advantage of being adapted to evolving knowledge about the products applied to the soil, thereby respecting the precautionary principle. Although it is always necessary to act upstream to reduce the load of contaminants entering the wastewater network, the use of the TCCI makes it possible to expand wastewater recycling while controlling potential risks.

The example of the agricultural use of SS and TWW from the Boumerdes WWTP is a good illustration of this. If only the total metal content in the spread products and in the soil is known, the calculation of the TCCI would result in a recommendation against agricultural spreading in the usual quantities due to high-risk values for Cr, Cu, Ni, and Zn. Given that we know the speciation of metals in the spread products and in the soils, as well as the pH of the soils, the calculation could be refined, and the recalculated TCCI values showed a low or moderate risk for all the metals. Thus, spreading under the usual conditions can be maintained. Other soil parameters affecting the mobility of metals, such as the content of clay, iron or manganese oxides, exchangeable cations, or organic matter, could be taken into account, when they are known, in the TCCI calculation for better risk assessment.

## Supplementary Information

Supplementary Material 1

## Data Availability

No datasets were generated or analysed during the current study.
